# Supplementary figures and images for: Linking pangenomes and metagenomes: the Prochlorococcus metapangenome
Source: PeerJ. 2018 Jan 25;6:e4320. doi: 10.7717/peerj.4320 (PMC5804319; doi:10.7717/peerj.4320)

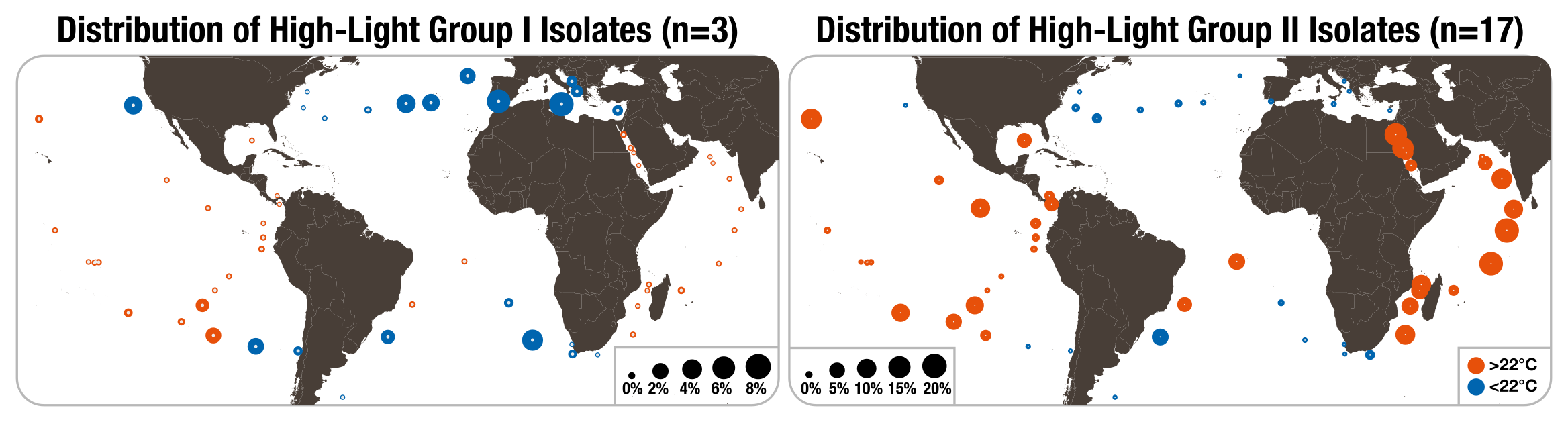

Supplement: Figure S1 — World maps describe the cumulative relative distribution of Prochlorococcus isolates from the clades HL-I (3 genomes) and HL-II (17 genomes) across 61 surface metagenomes. The size and color of dots varies as a function of relative distributions and temperature range (<22 °C versus >22 °C), respectively. [file peerj-06-4320-s001.png]

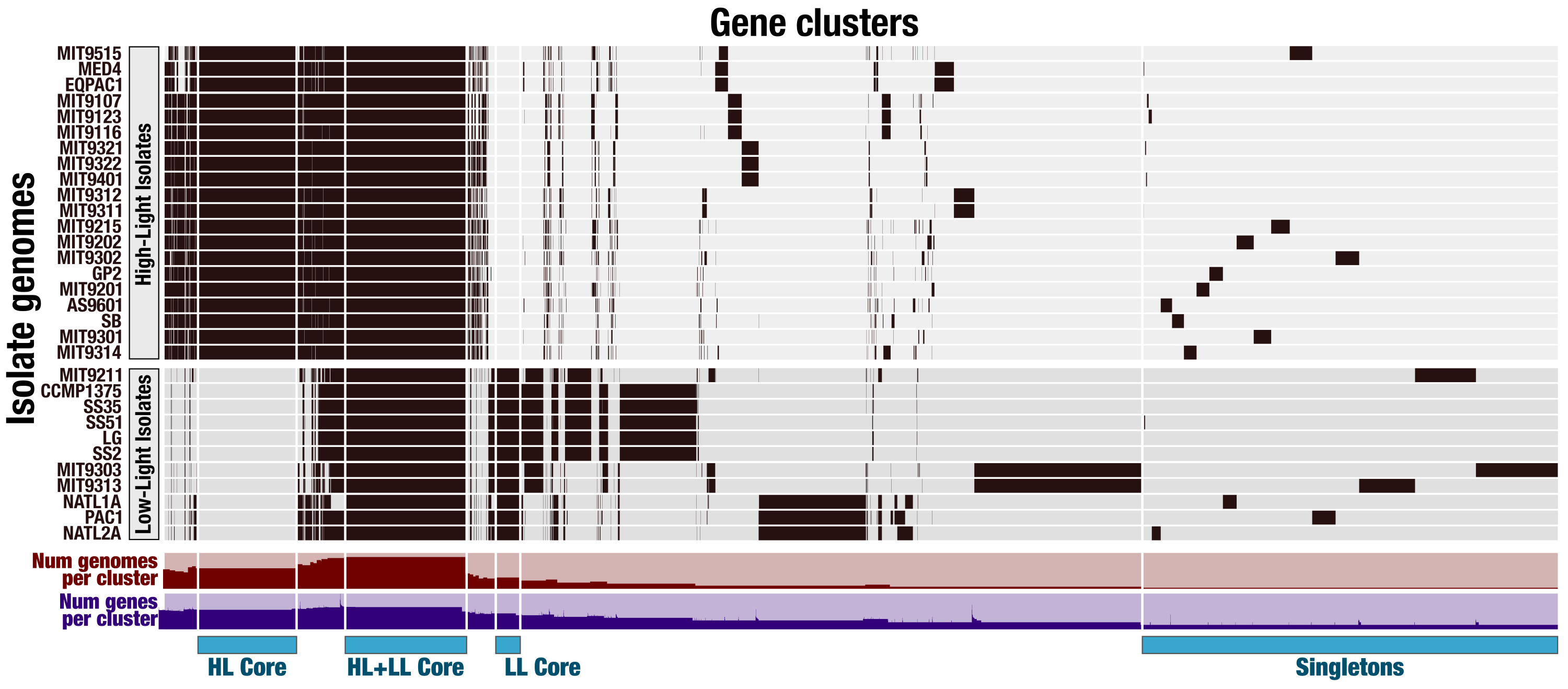

Supplement: Figure S2 — Each one of the 7,385 gene clusters contains one or more genes contributed by one or more isolate genomes. Bars in the 31 horizontal layers indicate the occurrence of gene clusters in a given isolate genome. Gene clusters are organized based on their distribution across genomes (i.e., gene clusters that co-occur in the same group of isolates are closer to each other), and genomes are organized based on gene clusters they share using Euclidian distance and ward ordination. The “HL + LL Core” selection corresponds to the clusters that contained genes from all genomes. The “LL Core” and “HL Core” selections correspond to gene clusters that contained genes characteristic to the LL- and HL-adapted genomes, respectively. The last selection (“Singletons”) corresponds to clusters that contained one or multiple genes from a single genome. [file peerj-06-4320-s002.png]

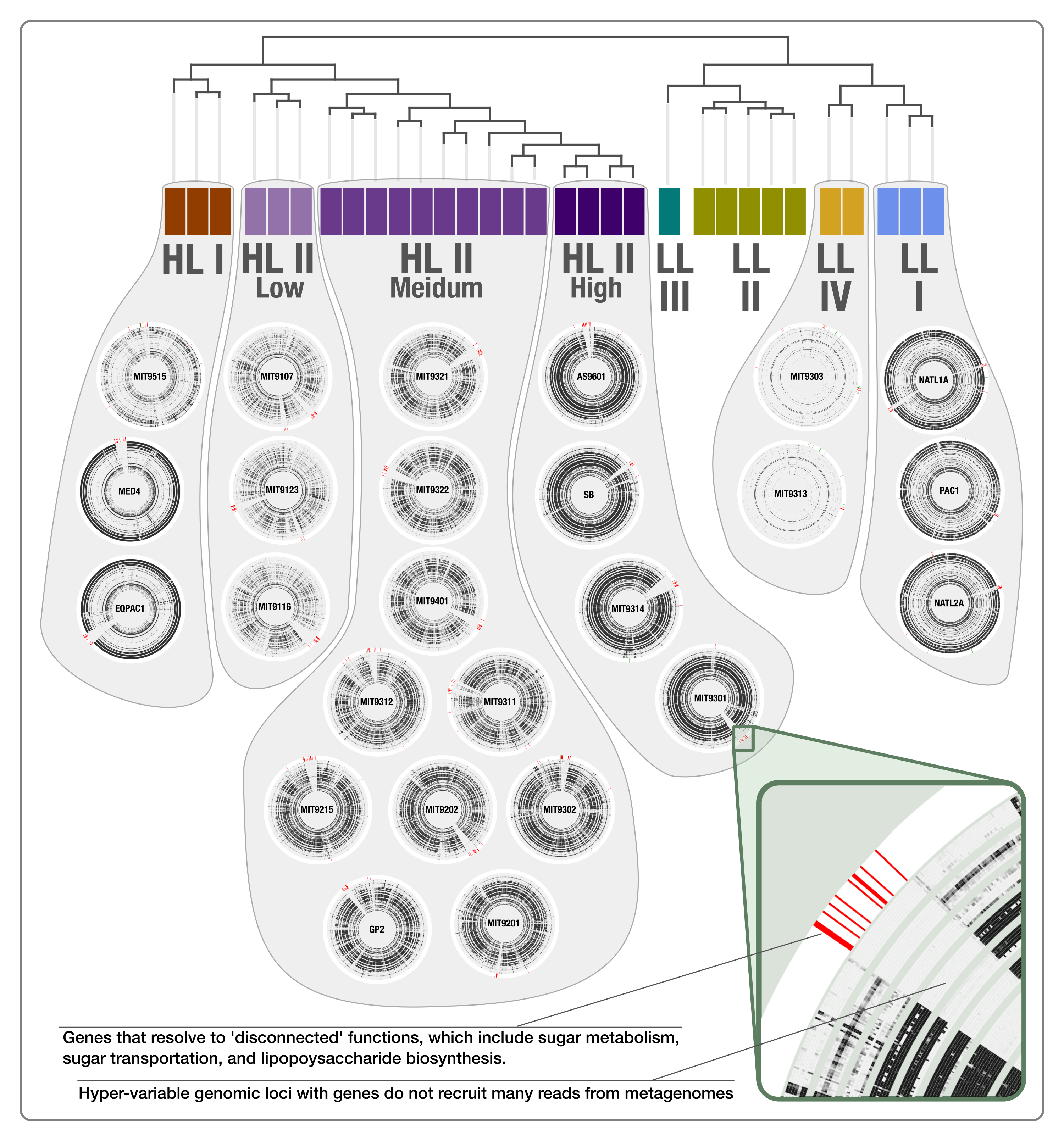

Supplement: Figure S3 — The figure displays the coordinates of genes corresponding to the 25 most environmental accessory functions across isolates genomes Prochlorococcus (red in the outer layers). Inner layers correspond to the 93 TARA Oceans metagenomes, organized by geographic regions similarly to Fig. 2. For each metagenome, black sections correspond to well covered genes while white sections correspond to genes with no read recruitment. Genomes are organized based on gene clusters similarly to Fig. 3. [file peerj-06-4320-s003.png]
